# Patient perspectives on incretin‐based weight loss medications and relationship with demographic factors

**DOI:** 10.1002/osp4.783

**Published:** 2024-08-06

**Authors:** Megan A. McVay, Wendy S. Moore, Francesca L. Wilkins, Jalen R. Jackson, Michael D. Robinson

**Affiliations:** ^1^ Department of Health Education & Behavior University of Florida Gainesville Florida USA; ^2^ Center for Integrative Cardiovascular and Metabolic Disease University of Florida Gainesville Florida USA; ^3^ Department of Medicine University of Florida Gainesville Florida USA

**Keywords:** education, glucagon‐like peptide 1(GLP‐1), obesity, pharmacologic therapy, weight loss

## Abstract

**Objective:**

Treatment of obesity has been transformed by the recent approval of incretin‐based therapies for weight loss (e.g., glucagon‐like peptide 1 agonist semaglutide), but little is known about patient perspectives on these medications.

**Methods:**

Between December 2023 and March 2024, healthcare patients from an academic medical center in the Southeast United States with Body Mass Index ≥30 kg/m^2^ completed a cross‐sectional online survey on attitudes toward incretin‐based medications.

**Results:**

Compared to patients with a bachelor's degree, those without a degree were less likely to be aware of incretin‐based pharmacotherapies (96% vs. 78%) and to have discussed pharmacotherapies with a doctor (43% vs. 27%) but had greater interest in using these pharmacotherapies (4.3 vs. 4.7). These pharmacotherapy‐related variables did not differ significantly according to gender, race, or financial security. Concerns about side effects, long‐term health risks, and potential for weight regain were highly endorsed and were associated with lower interest in using incretin‐based therapies and with some demographic factors. Patients reported high interest in lifestyle programs designed for individuals taking anti‐obesity medications.

**Conclusion:**

Demographic considerations, notably education level, should be factored into the strategy to promote equitable utilization of incretin‐based therapies, particularly as their accessibility expands.

## 1 INTRODUCTION

The treatment of obesity has been transformed by the food and drug administration approval of incretin‐based therapies (i.e., those targeting glucagon‐like peptide 1 (GLP‐1) and gastric inhibitory polypeptide receptors) for the treatment of obesity. Approved incretin‐based pharmacotherapies semaglutide (approved in 2021) and tirzepatide (approved in 2023) have resulted in mean weight loss of 12%–18% greater than placebo^1^, ^2^, ^3^, ^4^ and notable health improvements^5^, ^6^ in clinical trials.

The use of anti‐obesity pharmacotherapies has historically varied among different demographic groups. Prior classes of weight loss medications, as well as the use of GLP‐1s for diabetes, have had documented lower use among non‐White individuals^7^, ^8^ individuals with lower incomes,^9^ and men.^10^, ^11^, ^12^ Even in a setting where cost and access constraints are lower (i.e., the Veterans Affairs health care system), demographic disparities in use of GLP‐1s have been found.^8^ This suggests that patient and/or provider perceptions and behaviors related to these medications are relevant for understanding their uptake, as also observed in uptake of bariatric surgery.^13^, ^14^

While the use of incretin‐based therapies for obesity is currently limited due to cost and availability, accessibility is expected to increase over time. To understand the potential risk for inequitable uptake of these medications as access barriers diminish, it is important to consider how sociodemographic factors are related to medication experiences and attitudes. Thus, the aims of the current study are to characterize awareness and interest related to incretin‐based pharmacotherapy for weight loss among healthcare patients, and to examine how those differ across demographic characteristics of gender, race, ethnicity, educational attainment, and financial status. This study also examines perceptions of barriers and benefits to use of these medications overall and across different demographic characteristics.

## 2 METHODS

### 2.1 Participants and procedures

Patients in a large academic health system in the US Southeast were identified through a search of the health systems clinical database and invited to complete the survey via email. Eligibility criteria included age 18–85 years, self‐report Body Mass Index ≥30 kg/m^2^, and ability to read and understand English. Patients were excluded if they were currently undergoing radiation or chemotherapy for cancer, were pregnant, or failed to correctly answer survey validation items.

Eligible patients answered a series of questions about anti‐obesity medications and other weight loss approaches (only medication‐focused results reported herein) and were paid $15–20. Ethics approval to conduct this study was obtained from the University of Florida Institutional Review Board and informed consent was obtained.

### 2.2 Measures

Prior to the current study, survey items were tested in the target population with individual cognitive interviews (*n* = 3) to ensure understanding and expected interpretation of items.^15^ All items are available at the Open Science Foundation project website (https://osf.io/dze8b/?view_only=914814d4cf19475e9b0c0827140b121b).

**Demographic and weight‐related measures.** Patients reported demographic characteristics, including age, gender, race, ethnicity, and marital status. They also answered a question about financial security, allowing classification as low (difficulty paying their bills) or moderate‐to‐high (enough money to pay their bills) security.^16^ They self‐reported their weight and height and indicated if they had an obesity‐related comorbidity of type 2 diabetes, pre‐diabetes, heart disease, hypertension, dyslipidemia, or sleep apnea.

**Medication‐related variables.** Patients first reported past anti‐obesity medication use. Next, to assess awareness of incretin‐based medications, patients were provided a brief introduction to these medications and then asked, “Have you heard about these newer GLP‐1 medications (such as Wegovy, Ozempic, and Mounjaro) prescribed for weight loss?” Patients were also asked if they had ever spoken to a doctor about these medications. Interest in incretin‐based medications was measured using the mean of three items developed for this study (e.g., “I would like to learn more about GLP‐1 medications”), with instructions to assume the medications are covered by their insurance; the measure had strong internal consistency (*α* = 0.915).

Patients were asked the extent to which the following factors would be barriers to the use of incretin‐based medications for weight loss: concern about side effects, injecting medication with a needle, potential for long‐term risks, and risk of weight re‐gain upon stopping medication. These barriers were rated as “not at all a barrier” (1) to “major” barrier (4). Perceived benefits of medications were measured with a single item asking expectations of how much weight they would expect to lose in 6 months with these medications, with options ranging from 1 (“0–4 pounds”) to 4 (“20 or more pounds”). Patients were also asked how likely they would be to join a lifestyle program offered for medication users (1‐ very likely, 4‐very unlikely).

### 2.3 Statistical analyses

We conducted chi‐square goodness of fit tests to examine bivariate associations between demographic factors and medication‐related variables of awareness, use, and discussion with the doctor. We conducted multivariate linear regression with demographic factors (age, gender, race, ethnicity, and marital status, financial status, and presence of comorbidities) as predictors of interest in incretin‐based medications, and ordinal logistic regression models to predict endorsement of barriers to use. Spearman's rank order correlations were calculated for associations between medication interest and ratings of medication barriers and benefits. Analyses were conducted in R.

## 3 RESULTS

### 3.1 Participant characteristics

Out of 2171 patients emailed, 290 started the screening. Of these, 230 met initial eligibility criteria and started the main survey and 207 completed the survey. Due to failing the validation item, 17 (5.9%) were excluded, leaving a final sample size of *n* = 190. Table 1 shows patient characteristics.

**TABLE 1:** Participant characteristics.

	Total sample (*n* = 190)^a^
Age, years, M (SD)	51.3 (15.4)
BMI (kg/m^2^), M (SD)^a^	37.8 (6.6)
Gender, *n* (%)	
Women	123 (64.7%)
Men	67 (35.3%)
Education, *n* (%)	
Less than bachelor's	120 (63.2%)
Bachelor's or higher	70 (36.8%)
Race, *n* (%)	
Black/African American	51 (26.8%)
White	121 (63.7%)
Asian	2 (1.0%)
Native Hawaiian/Pacific Islander	4 (2.1%)
American Indian or Alaska native	0 (0%)
Other	13 (6.8%)
Ethnicity, *n* (%)	
Hispanic/Latino	30 (15.7%)
Non‐Hispanic/Latino	160 (84.2%)
Relationship status, *n* (%)	
Partnered	109 (57.4%)
Not partnered	81 (42.6%)
Financial security, *n* (%)	
Low	62 (32.6%)
Moderate‐to‐high	128 (67.4%)
Weight‐related comorbidity present	
Yes	155 (81.6%)
No	35 (18.4%)
Ever used any weight loss medication	
Yes	64 (33.7%)
No	126 (66.3%)
Ever used incretin‐based medication	
Yes	28 (14.7%)
No	162 (85.3%)

Abbreviation: BMI, Body Mass Index.

^a^ *N* = 186 for BMI (4 BMI values were implausible and removed from data set).

Experience and attitudes toward incretin‐based anti‐obesity medications overall and according to demographic factors are presented in Table 2. While 84% of patients overall were aware of incretin‐based anti‐obesity medications, awareness was lower among those without a bachelor's degree compared to those with a degree (77% vs. 96%) and among those without comorbidities compared to those with comorbidities (66% vs. 88%). Overall, 15% of patients reported having ever used an incretin‐based medication for weight loss, and 8% (*n* = 15) reported using one in the previous 6 months. One‐third of the patients reported that they had discussed these medications with their doctor. This discussion was more common for women than men (38% vs. 22%), for those with a bachelor's degree than those without (43% vs. 27%), and for those with comorbidities than those without (24% vs. 11%).

**TABLE 2:** Association of demographic factors with awareness, use and discussion with doctors of incretin‐based pharmacotherapy.

		Gender		Education		Race		Financial security		Comorbidities	
	Overall	Women (*n* = 123)	Men (*n* = 67)	Less than bachelors (*n* = 120)	Bachelors or higher (*n* = 70)	White (*n* = 121)	Black/African American (*n* = 51)	Low (*n* = 62)	High (*n* = 128)	Yes (*n* = 155)	No (*n* = 35)
Aware of incretin‐based pharmacotherapy											
Yes	160 (84%)	104 (85%)	56 (84%)	93 (77%)**	67 (96%)	105 (87%)	40 (78%)	50 (81%)	110 (86%)	137 (88%)*	23 (66%)
No	30 (16%)	19 (15%)	11 (16%)	27 (23%)	3 (4%)	16 (13%)	11 (22%)	12 (19%)	18 (14%)	18 (12%)	12 (34%)
Ever used incretin‐based pharmacotherapy											
Yes	28 (15%)	22 (18%)	6 (9%)	15 (13%)	13 (19%)	18 (15%)	8 (16%)	11(18%)	17 (13%)	27 (17%)	1 (3%)
No	162 (85%)	101 (82%)	61 (91%)	105 (88%)	57 (81%)	103 (85%)	43 (84%)	51(82%)	111 (87%)	128 (83%)	34 (97%)
Discussed incretin‐based pharmacotherapy with doctor											
Yes	62 (33%)	47 (38%)*	15 (22%)	32 (27%)*	30 (43%)	44 (36%)	15 (29%)	23 (37%)	39 (30%)	31 (24%)**	4 (11%)
No	128 (66%)	76 (62%)	52 (78%)	88 (63%)	40 (57%)	77 (64%)	36 (71%)	39 (63%)	89 (70%)	97 (76%)	31 (89%)

*p < 0.05, **p < 0.01, ***p < 0.001.

Mean ratings of interest in considering the use of incretin‐based anti‐obesity medications was 4.6 (SD = 1.3) on a 1–6 scale, suggesting a moderate‐to‐high overall interest. Interest was greater among those with less than a bachelor's degree and with comorbidities (see Table 3). Overall, barriers related to side effects (*M* = 2.9, SD = 1.1), long‐term risks (*M* = 3.1, SD = 1.0), and weight regain (*M* = 2.9, SD = 1.0) were endorsed at a moderate‐to‐high level, with concern about needle injections rated lower (*M* = 1.9, SD = 1.2). Concern about side effects was greater among those with a bachelor's degree, while concern about needle injections was greater for men, Black/African American respondents, and those without comorbidities (see Table 3). Higher endorsement of barriers was associated with lower interest in the medications (side effects: *ρ* −0.54; needles: *ρ* −0.39; long‐term risks: *ρ* −0.56; weight regain: *ρ* −0.36; all *p* < 0.001). Endorsement of barriers to medication use did not significantly differ between those who had and had not used incretin‐based medications in the past, with the exception of concern about needle injection, which was greater among never users (*χ* ^2^ = 16.0, *p* = 0.001). Anticipated weight loss outcomes with medication were distributed as follows: <10 lbs (20.5%), 10–19 lbs (35.3%), and ≥20 lbs (44.2%). Higher anticipated weight loss was positively associated with interest in medications (*ρ* = 0.25, *p* < 0.001) but was not associated with any demographic factors.

**TABLE 3:** Regression models for demographic factors and BMI as predictors of interest and barriers to incretin‐based pharmacotherapy for obesity.

	Medication interest^a^	Concern about side effects^b^	Concern about needle injection^b^	Concern about long‐term risks^b^	Concern about weight regain^b^	Expectations of weight loss^b^
BMI	0.01	0.005	−0.01	0.004	−0.03	0.04
Gender	0.01	−0.13	−0.91**	−0.07	−0.15	0.03
Race	−0.15	0.35	0.85*	0.20	0.41	−0.41
Education	0.74***	−0.65*	−0.07	−0.50	−0.61	0.23
Financial stress	0.07	−0.14	−0.28	−0.12	0.07	−0.21
Comorbidities	−0.67**	−0.61	−0.91*	−0.33	0.58	0.06

Abbreviation: BMI, Body Mass Index.

^a^ Linear regression.

^a^ Linear regression.

^b^ Ordinal logistic regression.

*p < 0.05, **p < 0.01, ***p < 0.001.

Most patients indicated they would be likely (38%) or very likely (51%) to enroll in a lifestyle program while taking an incretin‐based therapy, if offered. Men were less likely to be interested in a lifestyle program than women (34.3% vs. 60.1% very likely; *χ* ^2^ = 14.1, *p* = 0.003). Interest in lifestyle program did not differ between those with and without past use of incretin‐based therapies (64.3% vs. 48.7% very likely; *χ* ^2^ = 3.1, *p* = 0.37).

## 4 CONCLUSIONS

Little is known about perceptions of the use of incretin‐based anti‐obesity medications among the population eligible for these medications. Results of the current survey of health care patients indicate overall high awareness of and interest in these medications, but also high endorsement of barriers to medication use, which was associated with lower interest in the medications. Addressing barriers such as concern about side effects and long‐term risks may be an important step toward ensuring appropriate and equitable use of these medications. For example, it may be valuable to help patients understand the relative risks of medication use compared with the risks of untreated obesity.

Past research found demographic differences in the uptake of older classes of anti‐obesity medications and of GLP‐1s for diabetes.^7^, ^8^, ^9^, ^10^ In the current study, patients with lower educational attainment appear to have higher interest in incretin‐based anti‐obesity medications but may be at risk of lower access—even beyond current barriers related to cost and availability—due to less awareness of the medications and less frequent discussion of the medication with their healthcare providers. Additionally, given past findings of men being less likely to use anti‐obesity medications, it was notable that in the current study, interest in incretin‐based medications did not differ by gender. However, women were more likely to have discussed these medications with their doctor. Recent data indicates that nearly all doctors are aware of incretin‐based medications, with the majority reporting being very or extremely familiar with semaglutides.^17^ By being aware of the gap in patient knowledge about these medications and the likelihood of discussing these medications, doctors can play a key role in introducing these medications to all patients who have a clear indication for them.

Limitations of this study include recruiting individuals in a single healthcare system, low response rate, and being underpowered to detect smaller effects. Additionally, perceptions of the benefits of weight loss may vary depending on weight loss goals, which were not measured. Strengths of the study include recruiting a diverse sample, pilot testing of survey items, and use of survey validation items to enhance response quality.

As incretin‐based pharmacotherapies increase in accessibility, they are expected to transform the treatment of obesity. The current results indicate the importance of attending to differences in perceptions and experiences with these medications across demographic factors, especially education level and gender.

## CONFLICT OF INTEREST STATEMENT

The authors declare no conflicts of interest.
